# Is *Helicobacter pylori* infection a risk factor for non-alcoholic fatty liver disease in children?

**DOI:** 10.1007/s00431-024-05867-y

**Published:** 2024-11-27

**Authors:** Sana Barakat, Mohamed Abdel-Fadeel, Ola Sharaki, Mohamed El Shafei, Basant Elbanna, Aml Mahfouz

**Affiliations:** 1 Paediatric Department, Faculty of Medicine, Alexandria University, Champollion Street El-Khartoum Square, Azarita Medical Campus 21311, Alexandria, Egypt; 2 Clinical and Chemical Pathology Department, Faculty of Medicine, Alexandria University, Alexandria, Egypt; 3 Department of Diagnostic and Interventional Radiology, Faculty of Medicine, Alexandria University, Alexandria, Egypt

**Keywords:** Cytotoxin-associated gene A, *Helicobacter pylori*, Non-alcoholic fatty liver disease, Vacuolating cytotoxin A

## Abstract

*Helicobacter pylori* infection has been investigated as a potential risk factor for non-alcoholic fatty liver disease (NAFLD). Some studies suggest a possible link between the two conditions. The purpose of this study is to study the relationship between *H. pylori* infection and NAFLD in pediatrics and its relation to NAFLD grades. A case–control study to identify predictors of NAFLD and a comparative cross-sectional approach to determine factors affecting NAFLD grades were adopted. One hundred NAFLD children (ultrasound-based) and a control group of 100 non-NAFLD children were recruited. Both groups were evaluated by detecting *H. pylori* stool antigen. Immunoglobulin G antibodies to Cag A (cytotoxin-associated gene A), Vac A (vacuolating cytotoxin A), Gro EL (chaperonin Gro EL), HCPC (Helicobacter cysteine-rich protein C), and Ure A (Urease subunit A) were assessed in the serum of those with positive stool antigen. *H. pylori* infection was significantly higher in NAFLD children compared to the control group (64% versus 25%, *p*-value < .001). (NAFLD children showed higher Cag A and Vac A positivity (34, 10%) versus (2%, 0%) in the control group, respectively, *p*-value < .001). The regression model showed that *H. pylori* positivity (OR (odds ratio) = 5.021, 95% CI (confidence interval): 1.105–22.815), homeostatic model assessment of insulin resistance (Homa IR) (OR = 18.840, 95% CI: 3.998–88.789), waist percentile (OR = 1.184, 95% CI: 1.044–1.344), and triglycerides (OR = 1.029, 95% CI: 1.012–1.047) were predictors for NAFLD. Cag A positivity (OR = 2.740, 95% CI: 1.013–7.411) was associated with higher NAFLD grade (grade 2 fatty liver).

*Conclusions:* *H. pylori* infection could increase the risk of NAFLD in children. Triglycerides, waist circumference, and Homa IR are significant independent predictors of NAFLD.

**Table:** 

**What is Known:**
• *NAFLD has become one of the most common liver diseases among children because of the increased prevalence of pediatric obesity.* • *Dyslipidemia and insulin resistance play a central role in NAFLD pathogenesis.* • *NAFLD could be explained by the multiple-hit hypothesis. The gut microbiota is an important factor in this hypothesis (gut liver axis).*
**What is New:**
• *Helicobacter pylori infection could increase the risk of NAFLD in children.* • *H. pylori Cytotoxin-associated gene A (Cag A) positivity is associated with higher NAFLD grade.*

**Supplementary Information:**

The online version contains supplementary material available at 10.1007/s00431-024-05867-y.

## Introduction

Non-alcoholic fatty liver disease (NAFLD) has become one of the most common liver diseases among children because of the increased prevalence of pediatric obesity [1]. Its prevalence in Egypt reached 15.7% [2].

NAFLD is defined by the detection of hepatic steatosis by either imaging or histology after the exclusion of other causes of liver disease [3]. NAFLD is explained by the multiple-hit hypothesis. The gut microbiota effect represents an important factor in this hypothesis [4]. *Helicobacter pylori* (*H. pylori*) is now a growing health problem, and there is growing evidence of an association between *H. pylori* and NAFLD [5]. *H. pylori* can alter the gut microbiome and can increase gut permeability allowing the translocation of bacteria and inflammatory mediators into the portal circulation. This leads to the progression of NAFLD [5]. *H. pylori* also can penetrate and stay in the hepatocytes [6]. It is mainly dependent on cytotoxin-associated gene A (Cag A) and vacuolating cytotoxin A (Vac A) status which are the main determinants of *H. pylori* pathogenicity [6]. The relationship between *H. pylori* and NAFLD was studied in several research in adults, but to date, there is no clear answer to the questions regarding this. Moreover, because of a lack of adequate data on children, this research aimed to study this relationship among children attending the hepatology and nutrition clinic at Alexandria University Children’s Hospital (AUCH).

## Methods

### Study design

A case–control study design was adopted to identify significant predictors of NAFLD. Moreover, to determine factors that significantly affect NAFLD grades, a comparative cross-sectional approach was adopted. The study was conducted from November 2022 till November 2023.

### Study participants

All obese children aged 2–18 years attending the outpatient hepatology and nutrition clinic at AUCH were assessed for fatty liver by ultrasound. Children with dyspeptic symptoms were excluded. Children with fatty liver due to other causes rather than NAFLD (e.g., viral hepatitis, celiac disease, Wilson disease, history of steatotic drug intake as corticosteroids) were excluded from the study. One hundred obese children with fatty liver and a control group of 100 obese children with normal ultrasound (who attend the nutrition clinic at AUCH seeking nutritional advice for the obesity problem), matched for age and sex, were selected. Sample size was calculated using the Epi Inf-7 program [7] based on an expected prevalence of *H. pylori* infection of 68% among NAFLD (in similar research) [8] as compared to 41% [9] among healthy children, as reported in recent literature with a minimum of 77 children per group was required to test hypothesis at 5% level of significance and achieve 90% power. Informed consent was taken from the guardians of all children.

### NAFLD diagnosis

Because literature about transient elastography and magnetic resonance elastography is limited in pediatrics [10]. Ultrasound abdominal examination was done by a single expert radiologist using a 4.5 MHz convex probe (Mindray Diagnostic Ultrasound System; Shenzhen Mindray Bio-Medical Electronics, China). Fatty liver was graded as follows: grade 0, grade 1, grade 2, and grade 3 [11].

### History and examination

Demographic data and medical history were collected from both groups. Socioeconomic status scores were determined [12]. Examination namely anthropometric measurements, blood pressure measurement, and abdominal examination were performed. BMI, WC, SBP, and DBP percentiles were determined [13, 14].

### Laboratory investigations

About 1 g of stool from each child was collected in a 5 ml sample diluent to be assayed for *H. pylori* antigen using Enzyme Immunoassay Test (ELISA) technique kit (Eagle Biosciences, Inc., Amherst, NH, USA) [15]. There was no history of acid-suppressive medications or antibiotic intake within 4 weeks before testing.

Three milliliter of venous blood was collected in a BD vacutainer® tube from every child under an aseptic technique after overnight fasting. Serum was used for assay of lipid profile, liver enzymes, and homeostatic model assessment of insulin resistance (HOMA-IR) [16–19]. Part of the serum was kept at − 80 °C to be used for detection of *H. pylori* virulence factors in patients with positive stool antigen using Line immunoassay test kit for detection of IgG antibodies to cytotoxin-associated gene A (Cag A), vacuolating cytotoxin A (Vac A), chaperonin Gro EL (Gro EL), Helicobacter cysteine-rich protein C (HCPC), and Urease subunit A (Ure A) [20].

## Statistical analysis

Statistical analysis was conducted using IBM SPSS Statistics for Windows, version 25 (IBM Corp., Armonk, N.Y., USA). Numerical variables were presented as mean and standard deviation or median and range. Comparison between groups was done using independent samples t-test and ANOVA for normally distributed variables. On the other hand, Mann–Whitney and Kruskal–Wallis tests were used for not normally distributed variables. Categorical variables were represented as numbers and percentages and compared using the Chi-square test. A multivariable binary logistic regression analysis was used to identify significant predictors of NAFLD after controlling for confounding factors using only factors that showed statistical significance in the univariate model and might be a risk factor for NAFLD. Ordinal logistic regression analysis was conducted to test significant predictors of higher NAFLD grades. Analysis was done at a 5% level of significance.

## Results

The study was conducted on 100 obese NAFLD children (54 (54%) males and 46 (46%) females). The same number of obese children with no NAFLD (65 (65%) males and 35 (35%) females) served as the control group. Their demographic, clinical, and laboratory data are illustrated in Table 1.

**Table 1:** Comparison between cases and control group as regards clinical, biochemical, and radiological characteristics

	NAFLD (*n* = 100)	No NAFLD (*n* = 100)	*p*-value
Age (years) (mean ± SD)	10.557 ± 3.068	9.780 ± 3.107	.077
Sex (male/female) *n* (%)	54/46 (54/46%)	65/35 (65/35%)	.113
SES score (median and range)	43 (20–72)	44 (19–77)	.199
BMI percentile (mean ± SD)	98.895 ± 1.011	97.256 ± 1.460	.001*
Waist percentile (mean ± SD)	91.190 ± 5.506	77.820 ± 10.144	.001*
SBP percentile (mean ± SD)	70.420 ± 15.893	61.750 ± 13.178	.001*
DBP percentile (mean ± SD)	71.280 ± 16.910	62.500 ± 12.683	.001*
ALT (U/L) (median and range)	30 (8–190)	16 (7–36)	.001*
AST (U/L) (median and range)	29 (14–99)	23 (11–39)	.001*
GGT (U/L) (median and range)	25 (7–141)	9 (5–22)	.001*
TC (mg/dL) (mean ± SD)	171.650 ± 35.538	157.430 ± 32.937	.004*
LDL-C (mg/dL) (mean ± SD)	100.830 ± 35.972	94.084 ± 34.532	.178
HDL-C (mg/dL) (mean ± SD)	41.900 ± 7.390	47.209 ± 5.276	< .001*
TG (mg/dL) (median and range)	133 (30–358)	77 (33–242)	< .001*
Homa IR (mean ± SD)	4.741 ± 2.355	1.479 ± 0.454	< .001*
*H. pylori* + ve, *n* (%)	64 (64%)	25 (25%)	< .001*
Cag A, *n* (%)	34 (34%)	2 (2%)	< .001*
Vac A, *n* (%)	10 (10%)	0 (0%)	.036*
Gro EL, *n* (%)	7 (7%)	3 (3%)	.887
HCPC, *n* (%)	5 (5%)	2 (2%)	.976
Ure A, *n* (%)	2 (2%)	0 (0%)	.371
US Grading *n* (%)			
G 0 G 1 G2 G3	0 (0%) 48 (48%) 31 (31%) 21 (21%)	100 (100%) 0 (0%) 0 (0%) 0 (0%)	< .001*

*NAFLD* non-alcoholic fatty liver disease, *SES* socioeconomic status, *BMI* body mass index, *SBP* systolic blood pressure, *DBP* diastolic blood pressure, *ALT* alanine aminotransferase, *AST* aspartate aminotransferase, *GGT* gamma-glutamyl transferase, *TC* total cholesterol, *LDL-C* low-density lipoprotein cholesterol, *HDL-C* high-density lipoprotein cholesterol, *TG* triglyceride, *Homa IR* homeostatic model assessment of insulin resistance, *H. pylori* Helicobacter pylori, *Cag A* cytotoxin-associated gene A, *Vac A* vacuolating cytotoxin A, *GroEL* chaperonin GroEL, *HCPC* Helicobacter cysteine-rich protein C, *Ure A* Urease subunit A, *US* ultrasound, * means significant difference

Numerical variables were expressed as mean ± SD (standard deviation) or median and range. Categorical variables were expressed as numbers (% percentages)

Independent sample t-test or Mann- Whitney test was used for numerical variables. The Chi-square test was used for categorical variables

The present study showed that NAFLD children had statistically high (BMI, waist, SBP, DBP) percentiles, ALT, AST, GGT, TG, TC, and Homa IR. Also, it revealed that NAFLD children had statistically low HDL-C. NAFLD cases had a statistically increased* H. pylori*-positive infection (64% versus 25% in non-NAFLD subjects, *p*-value < 0.001). As regards *H. pylori* virulence factors, Cag A and Vac A positivity were statistically exhibited in the NAFLD group (34%, 10%) than in the non-NAFLD group (2%, 0%), respectively, with *p*-value < 0.05. Regarding NAFLD grading by ultrasound, 48% of the cases had grade 1, 31% had grade 2, and 21% had grade 3 fatty liver. There were no significant differences between NAFLD and non-NAFLD children regarding socioeconomic status (Table 1).

Table 2 shows the risk factors predicting NAFLD. Waist percentile, TG, Homa IR, and H pylori positivity were positive independent predictors for NAFLD after multivariate analysis. The overall model showed that 88.7% of NAFLD could be predicted by the significant factors in the model (*R*^2^ = 0.887).

**Table 2:** Univariate and multivariable binary logistic regression analysis to predict the risk of NAFLD

	Univariate		Multivariable-adjusted		
	OR (95%CI)	*P-*value	OR (95%CI)	*P*-value	
BMI percentile	1.200 (1.154–1.249)	< .001*	.872 (.416–1.829)	.718	*X*^2^=218.974
Waist percentile	1.030 (1.025–1.035)	< .001*	1.184 (1.044–1.344)	.009*	
SBP percentile	1.009 (1.005–1.014)	< .001*	.952 (.896–1.011)	.111	
DBP percentile	1.009 (1.005–1.013)	< .001*	1.035 (.972–1.102)	.285	Nagelkerke *R*^2^ = .887
TG	1.004 (1.003–1.004)	< .001*	1.029 (1.012–1.047)	.001*	
TC	1.003 (1.001–1.005)	.004*	1.004 (.975–1.033)	.804	
HDL- C	.972 (.963–.982)	< .001*	1.066 (.907–1.252)	.441	*P* ≤ .001*
Homa IR	1.159 (1.135–1.184)	< .001*	18.840 (3.998–88.789)	< .001*	
*H. pylori* infection	5.333 (2.899–9.811)	< .001*	5.021 (1.105–22.815)	.037*	

*BMI* body mass index, *SBP* systolic blood pressure, *DBP* diastolic blood pressure, *TG* triglyceride, *TC* total cholesterol, *HDL-C* high-density lipoprotein cholesterol, *Homa IR* homeostatic model assessment of insulin resistance, *H. pylori* Helicobacter pylori, *OR* odds ratio, *CI* confidence interval, * means significant difference

Table 3 compares the studied parameters of NAFLD cases and NAFLD grades by ultrasound. It showed that high (BMI, waist, SBP, DBP) percentiles, ALT, GGT, TG, Homa IR, and low HDL-C were significantly associated with higher NAFLD grades. In children with *H. pylori-*positive infection, Cag A positivity had the highest prevalence among cases with grade 2 fatty liver.

**Table 3:** Comparison between the studied parameters of NAFLD cases and NAFLD grades by ultrasound

	NAFLD grade 1 (*n* = 48)	NAFLD grade 2 (*n* = 31)	NAFLD grade 3 (*N* = 21)	*P*-value
BMI percentile (mean ± SD)	98.581 ± 1.190	99.088 ± .788	99.331 ± .557	.016*
Waist percentile (mean ± SD)	88.812 ± 6.456	93.000 ± 3.732	93.952 ± 2.011	< .001*
SBP percentile (mean ± SD)	64.437 ± 14.339	75.096 ± 14.337	77.190 ± 17.054	.001*
DBP percentile (mean ± SD)	64.166 ± 14.779	77.838 ± 14.769	77.857 ± 18.477	< .001*
ALT (U/L) (median and range)	28 (11–51)	30 (8–103)	48 (15–190)	.012*
AST (U/L) (median and range)	28 (16–51)	26 (14–53)	31 (18–99)	.136
GGT (U/L) (median and range)	18 (7–54)	30 (15–70)	38 (21–141)	< .001*
TG (mg/dL) (median and range)	109 (30–332)	141 (53–358)	167 (50–332)	.039*
TC (mg/dL) (mean ± SD)	166.500 ± 31.730	173.129 ± 35.971	181.238 ± 42.163	.276
HDL-C (mg/dL) (mean ± SD)	44.875 ± 6.561	39.741 ± 7.438	38.285 ± 7.436	< .001*
Homa IR (mean ± SD)	3.491 ± 1.598	4.906 ± 2.080	7.352 ± 2.005	< .001*
*H. pylori*, *n* (%)				
+ ve (*n* 64) -ve (*n* 36)	27 (42.187%) 21 (58.333%)	23 (35.937%) 8 (22.222%)	14 (21.875%) 7 (19.444%)	.257
*H. pylori* + ve Cag A,* n* (%)				
+ ve (*n* 34) -ve (*n* 30)	9 (26.470%) 18 (60%)	15 (44.117%) 8 (26.666%)	10 (29.411%) 4 (13.333%)	.024*

*BMI* body mass index, *SBP* systolic blood pressure, *DBP* diastolic blood pressure, *ALT* alanine aminotransferase, *AST* aspartate aminotransferase, *GGT* gamma-glutamyl transferase, *TG* triglyceride, *TC* total cholesterol, *HDL-C* high-density lipoprotein cholesterol, *Homa IR* homeostatic model assessment of insulin resistance, *H. pylori* Helicobacter pylori, *Cag A* cytotoxin-associated gene A, * means significant difference

Numerical variables were expressed as mean ± SD (standard deviation) or median and range. Categorical variables were expressed as numbers (% percentages). Kruskal–Wallis test or one-way ANOVA was used for numerical variables. The Chi-square test was used for categorical variables

Table 4 showed an ordinal logistic regression analysis to assess NAFLD parameters and their relation to NAFLD grades by ultrasound (using parameters that have significant differences when compared regarding NAFLD grades, as shown in Table 3). It showed that high waist percentile, DBP percentile, TG, and Homa IR were associated with higher NAFLD grades. Cag A positivity was associated with higher NAFLD grade (grade 2 fatty liver).

**Table 4:** Ordinal logistic regression analysis to assess NAFLD parameters and their relation to NAFLD grades by ultrasound

	Sig	OR (exp)	95% CI (exp)	
			Lower bound	Upper bound
BMI percentile	.502	1.262	.639	2.491
waist percentile	. 048*	1.164	1.001	1.352
SBP percentile	.543	.986	.942	1.031
DBP percentile	.047*	1.043	1.001	1.089
TG	.030*	1.009	1.001	1.017
HDL-C	.497	.970	.891	1.057
Homa IR	< .001*	1.995	1.526	2.606
Cag A + ve	.047*	2.740	1.013	7.411
Significance	*X*^2^ (8) = 82.495		*P* = < .001*	
Nagelkerke *R*^2^	.641			

*BMI* body mass index, *SBP* systolic blood pressure, *DBP* diastolic blood pressure, *TG* triglyceride, *HDL-C* high-density lipoprotein cholesterol, *Homa IR* homeostatic model assessment of insulin resistance, *Cag A* cytotoxin-associated gene A, *OR* odds ratio, *CI* confidence interval, * means significant difference

## Discussion

NAFLD is a growing health problem in children due to increasing obesity concerns and excess consumption of fructose [1]. With increasing NAFLD severity, hepatocyte injury can lead to fibrosis and cirrhosis. *H. pylori* is the most common organism infecting the gastrointestinal tract. Its prevalence in developed countries reaches around 20%, but in developing countries, it reaches up to 70% [21]. Recently, an association has emerged between NAFLD and *H. pylori* infection with a possible causal link between them. Furthermore, *H. pylori* infection could exacerbate NAFLD. This is explained by the effect of *H. pylori* in causing low-grade systemic inflammation and releasing inflammatory cytokines, which further affect lipid metabolism, cause gut dysbiosis, alter the intestinal barrier, and promote insulin resistance. Another mechanism is that cells infected with *H. pylori* may release extracellular vesicles, and *H. pylori* can release outer membrane vesicles, which can directly impact the liver and contribute to the progression of NAFLD [22]. Also, previous studies reported that *H. pylori* infection could be detected in the liver tissue of NAFLD subjects [6].

To the best of our knowledge, there is a lack of studies done on pediatrics. So, the current study investigated *H. pylori* infection in children suffering from NAFLD and its effect on NAFLD grades.

The present study showed an increased *H. pylori* infection prevalence in children suffering from NAFLD (64% of NAFLD children versus 25% of children with no NAFLD). Khalil AE et al. [23] reported 70% in NAFLD cases versus 30% in controls and Mostafa NR et al. [8] (68% versus 32% in non-NAFLD subjects).

In the current study, *H. pylori* infection was an independent predictor of NAFLD (OR 95% CI 5.021 (1.105–22.815)). A similar result was observed by Yan P et al. [24] (95% CI 1.02–1.79, OR 1.35, *p* = 0.036), Sumida Y et al. [25] (95% CI 1.111–7.644, OR 2.915, *p* = 0.03), and Mostafa NR et al. [8] (95% CI 1.967–16.130, OR 5.632, *p* = 0.001). This finding might have implications for both clinical practices such as screening and management strategies.

The results of the current study are not consistent with Valadares EC et al. [26] Variations in study designs, participant characteristics, geographic locations, and methodologies could contribute to conflicting results across different studies.

Cag A and Vac A are the main virulence of *H. pylori* pathogenicity. Cag A positive stains are more motile and capable of producing inflammatory cytokines, causing gut dysbiosis and increasing gut permeability [27, 28]. Also, studies showed that *H. pylori* could enter and stay in the hepatocytes which is mainly dependent on Cag A and Vac A status [6].

The current study showed increased Cag A and Vac A prevalence among NAFLD versus non-NAFLD children (34% versus 2% for Cag A and 10% versus 0.00% for Vac A, *p*-value < 0.001, 0.036, respectively).

Similar results were reported by Alvarez C et al. [29]who reported that Cag A and Vac A positivity were associated with a two-to-three-times increase in NAFLD prevalence (OR = 2.19, 95% CI 1.05–4.58 for Vac A and OR = 2.73, 95% CI 1.03–7.20 for Cag A).

The current study reported Cag A positivity was associated with high NAFLD grade (grade 2 fatty liver) (OR = 2.740 (95% CI, 1.013–7.411), *p*-value 0.047). Barreyro FJ et al. [30] studied the relationship between Cag A strain and NAFLD. They reported higher AST and FIB-4 values in cases with Cag A positive *H. pylori* infection. Contradictory to the results of the current study, Kang SJ et al. [31] reported that NAFLD was associated with Cag A negative *H. pylori* infection.

The current study showed that BMI, WC, blood pressure, liver enzymes, lipid profile, and Homa IR were higher significantly in children suffering from NAFLD. Khalil AE et al. [23] reported higher BMI and lipid profile in NAFLD subjects. Jin R et al. [32] reported higher BMI, ALT, AST, total cholesterol, and Homa IR in NAFLD children. Mostafa NR et al. [8] found higher BMI in NAFLD cases, but in contrast to the present study, they did not report a significant difference regarding liver enzymes and lipid profile compared to non-NAFLD cases.

In the current study, although the median of ALT and AST showed significant differences between NAFLD and non-NAFLD children, it was generally within the normal limit (30 vs. 16, 29 vs. 23 U/L, *p* = 0.001), respectively. Similarly, Yavuz Özer et al. [33] in their study on 155 obese children (6–18 years) reported that the median ALT and AST in NAFLD and non-NAFLD children were 28 vs. 17 and 25 vs. 19 U/L, *p* = 0.001, respectively. A previous study concluded that normal ALT does not exclude NAFLD [34].

The primary type of fat that builds up in the liver of individuals with NAFLD is triglyceride (TG). Triglyceride accumulation in the liver could be a cause (through direct effect or accumulation of lipotoxin) or a consequence of hepatic steatosis through hepatotoxin-mediated injury [35]. The current study revealed that TG was an independent predictor of NAFLD (OR = 1.029, 95% CI: 1.012–1.047). This was agreed by previous research [36, 37].

The current study has points of strength, and it investigated the link between *H. pylori* infection and NAFLD in children despite that previous studies were done on adults. Also, it studied the association between *H. pylori* virulence factors and their relationship to NAFLD grades.

### The limitation of the current study

- It is a single-center study.

- Liver biopsy is the gold standard in NAFLD diagnosis. However, being an invasive maneuver and it is not ethical to be performed in asymptomatic children. So, abdominal ultrasound was used to detect NAFLD. To eliminate interobserver variability, it was done by a single expert radiologist.

-Upper gastrointestinal endoscopy was not done after detection of *H. pylori* positivity by stool antigen because it is not ethical to be performed in asymptomatic children.

## Conclusion

*Helicobacter pylori* infection could increase the risk of NAFLD in children. Further multicenter research is needed for a better understanding of the potential relationship between *H. pylori* and NAFLD and its broader implications taking into consideration other confounders for *H. pylori* infection. Furthermore, triglycerides, waist circumference, and Homa IR are significant independent predictors of NAFLD.

## Supplementary Information

Below is the link to the electronic supplementary material.

Supplementary file1 (DOCX 221 KB)

## Data Availability

Data will be available upon request.

## Abbreviations

ALT: Alanine-aminotransferase
AST: Aspartate-aminotransferase
AUCH: Alexandria University Children’s Hospital
BMI: Body mass index
Cag A: Cytotoxin-associated gene A
DBP: Diastolic blood pressure
FBS: Fasting blood sugar
GGT: Gamma-glutamyl transferase
Gro EL: Chaperonin Gro EL
HCPC: Helicobacter cysteine-rich protein C
HDL-C: High-density lipoprotein cholesterol
Homa IR: Homeostatic model assessment of insulin resistance
*H. pylori*: *Helicobacter pylori*
LDL-C: Low-density lipoprotein cholesterol
NAFLD: Non-alcoholic fatty liver disease
SBP: Systolic blood pressure
TC: Total cholesterol
TG: Triglyceride
Ure A: Urease subunit A
Vac A: Vacuolating cytotoxin A
WC: Waist circumference
