# Supplementary material for: Is Helicobacter pylori infection a risk factor for non-alcoholic fatty liver disease in children?
Source: Eur J Pediatr. 2024 Nov 27;184(1):47. doi: 10.1007/s00431-024-05867-y (PMC11602778; doi:10.1007/s00431-024-05867-y)
Supplement: Supplementary file 1 — Supplementary file1 (DOCX 221 KB) [file 431_2024_5867_MOESM1_ESM.docx]

**European Journal of Pediatrics**

**Is Helicobacter pylori infection a risk factor for non-alcoholic fatty liver disease in children?**

Sana Barakat ^1^, Mohamed Abdel-Fadeel ^1^, Ola Sharaki ^2^, Mohamed El Shafei ^3^, Basant Elbanna^1*^, Aml Mahfouz ^1^

^1^ Paediatric Department, Faculty of Medicine, Alexandria University, Alexandria, Egypt `

^2^ Clinical and Chemical Pathology Department, Faculty of Medicine, Alexandria University, Alexandria, Egypt

^3^ Department of Diagnostic and Interventional Radiology, Faculty of Medicine, Alexandria University, Alexandria, Egypt

* Corresponding author: Paediatric Department, Faculty of Medicine, Alexandria University,

Champollion Street El-Khartoum Square, Azarita Medical Campus 21311, Alexandria, Egypt. +2001283247238
E-mail address: b_abdelazim14@alexmed.edu.eg

ORCID ID: <https://orcid.org/0009-0009-8493-9309>


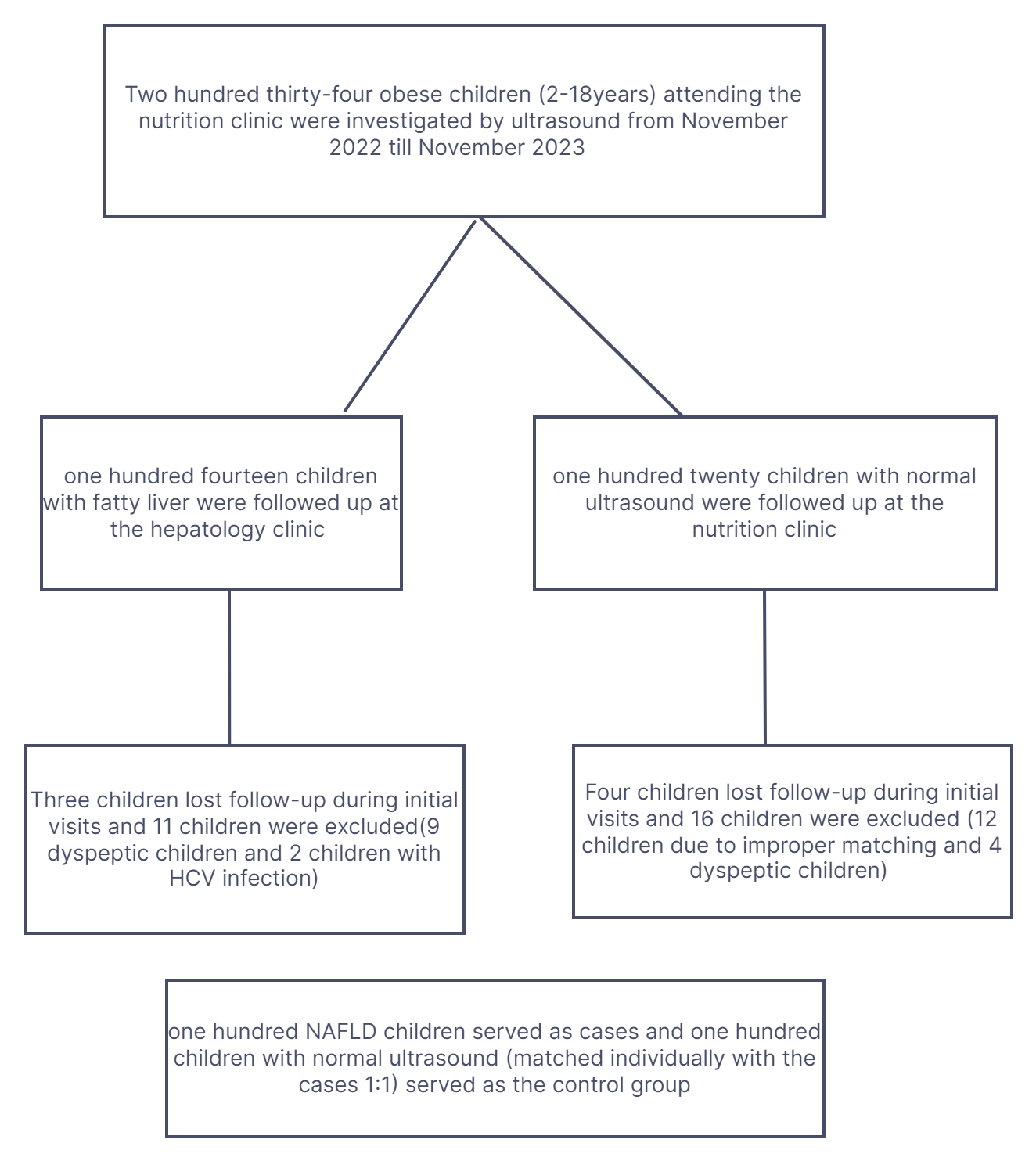


Figure 1. Flow chart of the study participants.
HCV: hepatitis c virus, NAFLD: Non-alcoholic fatty liver disease
